# Linking Adverse Childhood Experiences and Other Risk Factors to Subjective Cognitive Decline in an Aging Population

**DOI:** 10.5888/pcd20.230182

**Published:** 2023-12-21

**Authors:** Hailey Voyer, Semra Aytur, Nicole Tanda

**Affiliations:** 1 The Dartmouth Institute for Health Policy and Clinical Practice, Dartmouth College, Hanover, New Hampshire; 2 University of New Hampshire, Health Management and Policy, Durham, New Hampshire

## Abstract

**Introduction:**

The Centers for Disease Control and Prevention’s Healthy Brain Initiative (HBI) encourages an interdisciplinary approach to addressing the burden of subjective cognitive decline (SCD) among the aging US population as that population continues to increase. Our study is one of the first to evaluate associations between SCD and adverse childhood experiences (ACEs) and other modifiable risk factors to support implementation of the initiative.

**Methods:**

We used multivariate logistic regression to assess data from the 2020 Behavioral Risk Factor Surveillance System survey to evaluate associations between SCD and ACEs scores and sociodemographic, behavioral, and clinical risk factors. Models were weighted to account for the complex survey design.

**Results:**

Approximately 8.1% of survey respondents reported experiencing SCD within the past 12 months. Adjusted regression analysis showed that conditions such as depression (AOR, 2.85; 95% CI, 2.29–3.55), arthritis (AOR, 1.30; 95% CI, 1.05–1.60), and diabetes (AOR, 1.33; 95% CI, 1.05–1.68) were significantly associated with SCD. SCD was also associated with experiencing more than 3 falls per year (AOR, 2.95; 95% CI, 2.13–4.09), sleeping more than 9 hours per night (AOR, 2.06; 95% CI, 1.37–3.09), and physical inactivity (AOR, 1.32; 95% CI, 1.03–1.68). Two or more ACEs also significantly increased the odds of SCD (AOR, 1.69; 95% CI, 1.36–2.10).

**Conclusion:**

Findings from our study can be used to inform policy, environment, and systems change efforts aimed at addressing modifiable risk factors to support healthy aging. The role of ACEs as determinants of brain health across the life course should also be considered in the design of clinical and community-based interventions.

**Summary**

**What is already known on this topic?**

More than 16 million US adults live with some form of cognitive impairment, costing $206 billion in annual Medicare and Medicaid payouts. Previous studies have identified risk factors associated with subjective cognitive decline (SCD), such as physical inactivity, hypertension, diet, and smoking status.

**What is added by this report?**

Ours is among the first studies to examine the relationships between SCD and adverse childhood experiences along with other risk factors by using data from the 2020 Behavioral Risk Factor Surveillance System survey.

**What are the implications for public health practice?**

We identify modifiable SCD risk factors across the life course while providing examples of policy, environment, and systems changes that support implementation of the Centers for Disease Control and Prevention’s Healthy Brain Initiative.

## Introduction

In recent years, the aging population in the United States has increased following the baby boom of the 1940s to 1960s and a rising life expectancy (1). More than 16 million US adults are living with some form of cognitive impairment, costing $206 billion in annual Medicare and Medicaid payouts alone (2,3). From 1996 to 2014, the prevalence of cognitive impairment increased annually among adults aged older than 50 years by an average of 0.7% for women and 1.0% for men (4).

In 2005, the Centers for Disease Control and Prevention (CDC) established the Healthy Brain Initiative (HBI), which uses an interdisciplinary approach to address the challenges associated with cognitive decline and to promote overall brain health (5). As part of this initiative, a subjective cognitive decline (SCD) module was added to the Behavioral Risk Factor Surveillance System (BRFSS) survey in 2011 (6). Data from this module provide information on the burden of SCD, as well as risk and protective factors (6).

The objective of our study was to examine sociodemographic, behavioral, and health-related risk factors for SCD by using recent BRFSS data to support the implementation of HBI through changes to clinical practice and policy. We used the lens of the socioecological model to explore how changes at multiple levels (individual, interpersonal, community, organizational, environmental, and policy) may be used to address SCD and dementia as a complex systems issue (7).

Previous studies have used BRFSS data to identify risk factors associated with SCD, such as physical inactivity, hypertension, diet, and smoking status (8,9). Felitti et al extensively described a dose–response relationship between adverse childhood experiences (ACEs) and various health risk behaviors and chronic diseases in adulthood (10). Our study aimed to expand on prior literature by examining associations between ACEs and other risk factors for SCD to inform prevention initiatives.

We used data from the 2020 BRFSS survey to examine the independent relationship between ACEs and SCD above and beyond other known risk factors (11). We provide examples of policy, environment, and systems changes that can address risk factors across the life course in support of HBI implementation.

## Methods

### Study sample

BRFSS is a cross-sectional telephone survey collected at the state level that asks US residents questions about health-related risk behaviors, chronic health conditions, and preventive services (12). Participants are noninstitutionalized adults aged 18 years or older located in all 50 states, the District of Columbia, and 3 US territories (12). States are required to ask a set of core component questions and may choose to add optional modules, including the SCD and ACEs modules (12). In 2020, the average BRFSS response rate was 47.9%, comparable to other national telephone surveys (13). A total of 103,610 participants were included in our study from the 18 states that opted to include the SCD common module. Five hundred and forty-seven survey participants responded “don’t know/not sure” or refused to answer the first SCD screening question, 30,306 were excluded for being under the age of 45, and 8,905 were not asked all the SCD questions or had missing data (n = 63,852). Only 9 states participated in both the SCD and ACEs modules, and 22,434 participants answered all 11 ACEs questions. Our final analytic sample consisted of 17,042 observations for which no data were missing for any variable in the model. CDC publishes a list of modules used, by state (14).

### Measures

Our primary outcome of interest was SCD. Survey respondents were asked if they had “experienced confusion or memory loss that is happening more often or is getting worse” in the past 12 months (15). If a participant responded affirmatively, they were identified as having SCD and were then asked a series of 5 additional questions regarding their level of difficulty with day-to-day activities, whether they needed help with these activities, whether they were able to get help when needed, whether SCD interfered with socialization, and whether they had discussed their confusion or memory loss with a clinician.

Guided by prior research, we examined a total of 11 behavioral, clinical, and environmental factors in the 2020 BRFSS that were hypothesized to increase risk of SCD (8,11). These included having 2 or more ACEs; being a current or former smoker; physical inactivity; having diabetes, obesity, arthritis, coronary heart disease (CHD), stroke, or depression; having a history of recurrent falls; and the average number of hours of sleep per night (16).

Binary variables were coded as either “yes” or “no”. For variables where the survey question was asked on a Likert scale, responses of “always,” “usually,” or “sometimes” were coded as yes, and responses of “rarely” or “never” were coded as no, to be consistent with prior literature (8).

Other variables were derived and coded as follows:

**ACEs score.** Eleven questions (Box) regarding ACEs were converted into a summed ACEs score variable. The median ACEs score was 2. A binary variable representing an ACEs score of 2 or more was then used as a cut point for analysis.

**Box. Behavioral Risk Factor Surveillance System: Adverse Childhood Experiences Module Questions**

1. Did you live with anyone who was depressed, mentally ill, or suicidal?

2. Did you live with anyone who was a problem drinker or alcoholic?

3. Did you live with anyone who used illegal street drugs or who abused prescription medication?

4. Did you live with anyone who served time or was sentenced to serve time in a prison, jail, or other correctional facility?

5. Were your parents separated or divorced?

6. How often did your parents or adults in your home ever slap, hit, kick, punch, or beat each other up?

7. Not including spanking, (before age 18) how often did a parent or adult in your home ever hit, beat, kick, or physically hurt you in any way?

8. How often did a parent or adult in your home ever swear at you, insult you, or put you down?

9. How often did anyone at least 5 years older than you or an adult ever touch you sexually?

10. How often did anyone at least 5 years older than you or an adult try to make you touch them sexually?

11. How often did anyone at least 5 years older than you or an adult force you to have sex?

**Obesity.** Obesity was defined as a body mass index (weight in kg divided by height in m^2^) greater than or equal to 30.

**Falls.** The number of falls in the last year were grouped into 3 categories: 1) none, 2) between 1 and 3 falls, and 3) more than 3 falls.

**Sleep.** Hours of sleep per night were grouped into 3 categories based on prior research (16): 1) short duration (<7 h/night), 2) adequate sleep (7–9 h/night), and 3) long duration (>9 h/night).

**Physical inactivity.** Physical inactivity was defined as no physical activity in the last 30 days, other than that associated with the respondent’s regular job.

Five sociodemographic variables were also analyzed. These were race or ethnicity (Non-Hispanic White, Non-Hispanic Black, Hispanic, or Other Non-Hispanic), age (<65 y vs ≥65 y), education (high school or less, some college or technical school, and graduated from college or technical school), sex (male or female), and poverty status (at or below the federal poverty level of $25,000 annual income for a family of 3). The BRFSS survey contains de-identified, publicly available data that did not meet the definition of research involving human subjects and thus did not require institutional review board review.

### Statistical analyses

We used the SAS 9.4 statistical program (SAS Institute Inc) for all analyses. Appropriate sampling weights were applied to all analyses. BRFSS uses a 3-tiered weighting system to account for differences in probability of selecting geographic strata, density of telephone numbers in a given block, and the number of adults who use a particular telephone number (17). This system serves as a blanket adjustment for noncoverage of an area and nonresponse (17). We conducted descriptive univariate and bivariate analyses first, calculating frequencies and prevalence rates of risk factors to compare respondents with and without SCD. We used Wald χ^2^ tests to calculate associated *P* values.

Next, we used the SAS SURVEYLOGISTIC procedure to conduct multivariate modeling. The first model examined associations between SCD and potential demographic, behavioral, and health-related risk factors except ACEs. A second model assessed the associations between SCD and all risk factors, including ACEs. Adjusted odds ratios (AORs) and 95% CIs were calculated. Multicollinearity was assessed by examining variance inflation factors and correlation coefficients. A factor of less than 10 and a correlation coefficient of less than 0.8 was observed, suggesting that multicollinearity was not a major concern in the analysis.

## Results

Of the 63,852 respondents who participated in the SCD module, 5,443 (8.1%, percentage weighted) reported having experienced SCD in 2020. Of these, 41.2% reported having given up day-to-day household activities or chores because of their symptoms, and 38.0% reported needing assistance with these day-to-day activities (Table 1). Of the 38.0% who reported needing assistance, 87.4% said they were able to get the help they needed from family members or friends. Additionally, 35.9% of respondents with SCD said their confusion and memory loss had interfered with their ability to engage in social activities outside the home. Less than half (45.6%) of respondents with SCD had discussed their symptoms with their health care provider.

**Table 1:** Prevalence of Functional Difficulties and Other Factors Related to Subjective Cognitive Decline (SCD) Among Participants (N = 5,729), Behavioral Risk Factor Surveillance System Survey, 2020^a^

SCD-Related factors (N = 5,729)	Prevalence, %	
	Yes^b^	No^c^
Have you given up day-to-day household activities or chores?	41.2	58.8
Do you need assistance with these day-to-day activities?	38.0	62.0
Are you able to get the help you need with day-to-day activities?	87.4	12.6
Has confusion or memory loss interfered with your ability to engage in social activities outside the home?	35.9	64.1
Have you discussed your SCD symptoms with a health care professional?	45.6	54.4

a Taken from respondents to the Subjective Cognitive Decline module.

b Participant answered “always,” “usually,” or “sometimes.”

c Participant answered “rarely” or “never.”

### Demographics

Compared with those without SCD, respondents who self-reported SCD were more likely to be aged 65 years or older, have incomes below the federal poverty level, and have a lower education level (Table 2).

**Table 2:** Demographic, Health, and Behavioral Characteristics of Survey Participants Who Did And Did Not Report Subjective Cognitive Decline (SCD), Behavioral Risk Factor Surveillance System Survey, 2020^a^

Characteristics	Without SCD, (n = 20,441), n (%)^b^	With SCD, (N = 1,993), n (%)^b^	*P* value^c^
**Demographics**			
**Sex**			
Male	9,052 (47.0)	907 (46.0)	.65
Female	11,389 (53.0)	1,086 (54.0)	
**Age ≥65 years**	10,573 (42.2)	1,166 (48.2)	.004
**Annual income below federal poverty level^d^**	3,574 (24.0)	695 (44.4)	<.001
**Education**			
High school or less	5,711 (37.9)	736 (48.1)	<.001
Attended college	6,116 (33.5)	646 (33.3)	
College Graduate	8,548 (28.6)	607 (18.6)	
**Race**			
White, Non-Hispanic	14,338 (69.9)	1,441 (70.6)	.48
Black, Non-Hispanic	1,093 (6.4)	109 (6.4)	
Hispanic	1,366 (12.7)	147 (14.0)	
Other, Non-Hispanic	3,216 (11.0)	259 (8.9)	
**Health conditions**			
Obesity	5,674 (32.5)	628 (37.9)	.008
Diabetes	3,084 (16.3)	508 (28.9)	<.001
Depression	2,853 (14.5)	827 (46.1)	<.001
Arthritis	7,529 (37.8)	1,170 (58.5)	<.001
Stroke	934 (4.8)	248 (13.2)	<.001
Coronary heart disease	1,266 (6.6)	287 (14.1)	<.001
**Behavioral**			
**Smoking**			
Nonsmoker	11,624 (54.6)	819 (40.4)	<.001
Former Smoker	6,396 (32.0)	775 (36.3)	
Current Smoker	2,296 (13.4)	391 (23.3)	
**Physical inactivity^e^**	15,522 (73.0)	1,238 (55.8)	<.001
**Falls (last 12 mo)**			
None	15,416 (77.3)	953 (49.3)	<.001
1–3	4,126 (19.3)	695 (33.9)	
≥4	703 (3.4)	298 (16.8)	
**Sleep (h/night)**			
<7	5,938 (32.1)	770 (45.3)	<.001
7–9	13,596 (63.9)	1,020 (45.2)	
>9	737 (4.0)	166 (9.4)	
**ACE score^f^**			
0–1	13,633 (65.2)	948 (41.6)	<.001
≥2	6,808 (34.8)	1,045 (58.4)	

Abbreviation: ACE, adverse childhood event.

a Taken from respondents to the Subjective Cognitive Decline module.

b Values calculated by using appropriate weighting system and by restricting observations to those for which ACEs is not missing.

c *P* value calculated by using Wald χ^2^ test.

d Annual income below $25,000 for a family of 3.

e Physical inactivity defined as participant reporting no physical activity within the past 30 days.

f Score reflects the number of “yes” answers to the eleven ACEs questions for an individual.

In both the intermediate (AOR,1.46; 95% CI, 1.14–1.86) and final (AOR, 1.44; 95% CI, 1.13–1.84) models, having an income below the federal poverty level was associated with increased odds of reporting SCD compared with having an income above the federal poverty level (Table 3). Similarly, being aged 65 years or older was significant in the intermediate model (AOR, 1.35; 95% CI, 1.09–1.62) and after adjustment for ACEs in the final model (AOR, 1.45; 95% CI, 1.17–1.79). Race, sex, and education level were not significant risk factors for SCD in any model.

**Table 3:** Crude and Adjusted Odds Ratios for the Association Between Subjective Cognitive Decline and Adverse Childhood Experiences (ACEs) and Demographic, Health, and Behavioral Risk Factors, Participants (N = 17,042), Behavioral Risk Factor Surveillance System Survey, 2020^a^

Characteristics (N = 17,042)	Crude, OR (95% CI)^b^	Intermediate model, AOR (95% CI)^c^	Fully adjusted model, AOR (95% CI)^d^
**ACEs score of ≥2**	2.63 (2.23–3.11)	Not applicable	1.69 (1.36–2.10)^e^
**Health conditions**			
Obesity	1.27 (1.06–1.51)	0.92 (0.73–1.14)	0.89 (0.71–1.11)
Diabetes	2.09 (1.73–2.52)	1.33 (1.05–1.68) f	1.33 (1.05–1.68) f
Depression	5.03 (4.23–5.99)	3.03 (2.45–3.76) e	2.85 (2.29–3.55) e
Arthritis	2.32 (1.95–2.75)	1.31 (1.07–1.61) f	1.30 (1.05–1.60) f
Stroke	3.03 (2.36–3.88)	1.29 (0.94–1.77)	1.28 (0.93–1.76)
Coronary heart disease	2.34 (1.83–2.98)	1.36 (1.01–1.82) f	1.37 (1.02–1.84) f
**Physical inactivity**	2.14 (1.80–2.54)	1.30 (1.02–1.65) f	1.32 (1.03–1.68) f
**Smoking**			
Non-smoker	1.0 [Reference]		
Former smoker	1.54 (1.28–1.85)	1.15 (0.92–1.45)	1.08 (0.85–1.36)
Current smoker	2.35 (1.88–2.95)	1.26 (0.94–1.68)	1.18 (0.88–1.58)
**No. of falls in last 12 months**			
None	1.0 [Reference]		
1–3	2.77 (2.30–3.33)	1.72 (1.38–2.15) e	1.68 (1.34–2.10) e
≥4	7.71 (5.89–10.12)	3.17 (2.29–4.38) e	2.95 (2.13–4.09) e
**Sleep (h/night)**			
<7	2.00 (1.68–2.38)	1.39 (1.12–1.73) g	1.35 (1.08–1.68) g
7–9	1.0 [Reference]		
>9	3.33 (2.40–4.61)	2.09 (1.40–3.13) e	2.06 (1.37–3.09) e
**Sex**			
Male	1.0 [Reference]		
Female	1.04 (0.88–1.23)	0.85 (0.69–1.06)	0.84 (0.67–1.04)
**Race**			
White, Non-Hispanic	1.0 [Reference]		
Black, Non-Hispanic	1.0 (0.68–1.47)	0.92 (0.52–1.63)	0.89 (0.50–1.61)
Hispanic	1.09 (0.81–1.47)	1.33 (0.91–1.94)	1.31 (0.90–1.91)
Other, Non-Hispanic	0.81 (0.62–1.04)	0.86 (0.61–1.19)	0.85 (0.61–1.20)
**Age** ≥**65 years**	1.27 (1.08–1.50)	1.35 (1.09–1.62) g	1.45 (1.17–1.79) e
**Annual income below federal poverty level**	2.54 (2.11–3.05)	1.46 (1.14–1.86) g	1.44 (1.13–1.84) g
**Education**	0.73 (0.66–0.80)	0.93 (0.82–1.07)	0.93 (0.82–1.07)

a Taken from respondents to the Subjective Cognitive Decline module.

b Crude and intermediate odds ratios calculated by restricting observations to those for whom ACEs score is not missing.

c Adjusted for all variables in the table except for ACEs score.

d Adjusted for all variables in the table.

e Significant at *P* < .001.

f Significant at *P* < .05.

g Significant at *P* < .01.

### Health conditions

Respondents who self-reported SCD were more likely than those without to have any of the 6 health conditions measured (Table 2). After adjusting for ACEs in the final model, respondents who reported having arthritis were 30% more likely to have SCD (AOR, 1.30; 95% CI, 1.05–1.60) as were those who reported having depression, who were almost 3 times as likely to report SCD (AOR, 2.85; 95% CI, 2.29–3.55) (Table 3) as those without either condition. Having diabetes (AOR, 1.33; 95% CI, 1.05–1.68) or CHD (AOR, 1.37; 95% CI, 1.02–1.84) were also associated with an increased risk of SCD after adjusting for ACEs in the final model. Having had a stroke or having obesity were insignificant in the final model.

### Health behaviors

Compared with those without SCD, those with SCD were more likely to report behavioral risk factors (Table 2). Of the 4 health behaviors examined, 3 were significantly associated with increased odds of reporting SCD after adjusting for ACEs in the final model (Table 3). Both insufficient and excessive sleep habits resulted in increased odds of reporting SCD: those sleeping less than 7 hours a night had 1.35 times the odds (AOR, 1.35; 95% CI, 1.08–1.68) and those sleeping more than 9 hours a night had more than 2 times the odds (AOR, 2.06; 95% CI, 1.37–3.09) of reporting SCD compared with those who slept between 7 and 9 hours a night in the final model. Repeated falls were strongly associated with SCD in the final model; respondents who had between 1 and 3 falls in the last year were 68.0% more likely (AOR, 1.68; 95% CI, 1.34–2.10) to report SCD than those who reported no falls, and those with more than 4 falls in the last year were almost 3 times more likely (AOR, 2.95; 95% CI, 2.13–4.09). Physical inactivity also increased a person’s odds of SCD by 32.0% (AOR, 1.32; 95% CI, 1.03–1.68) in the final model. Being a former or current smoker was not a significant risk factor for SCD in either model.

### Adverse childhood events

In fully adjusted models that included ACEs controlling for other risk factors, respondents who reported 2 or more ACEs had 1.69 times greater odds of reporting SCD (AOR, 1.69; 95% CI, 1.36–2.10) compared with those who reported fewer than 2 ACEs (Table 3).

## Discussion

The literature examining risk factors for SCD has progressed in recent years to include consideration of ACEs as well as other exposures across the life course (11). Our study explored relationships between ACEs, SCD, and other risk factors by using 2020 BRFSS data collected at the height of the COVID-19 pandemic. In 2020, 8.1% of adults participating in the BRFSS SCD module reported having experienced SCD. Compared with prior years, a lower prevalence of SCD was self-reported in 2020 (18). In multivariate models, the most significant predictors of SCD were a history of depression, repeated falls, physical inactivity, and sleeping more than 9 hours per night. These modifiable risk factors could be managed appropriately if disclosed to a health care provider. For example, physical inactivity was associated with a 32.0% increased risk of SCD, consistent with prior research (9). Also consistent with prior studies, chronic conditions such as diabetes, arthritis, and CHD were found to be significant predictors of cognitive decline (1,8). Prior studies also found that adults with more than 1 comorbid chronic disease were more likely to have associated functional difficulties (1). In turn, worsening memory, as seen in SCD, makes managing these illnesses more difficult (1). Fewer than half of the respondents in the present study reported that they had discussed their SCD symptoms with a health care provider, comparable to prior years (18). This suggests an opportunity to educate both patients and clinicians about the importance of discussing SCD, reducing stigma, and framing SCD in the context of general wellness.

Lifestyle medicine is an evidence-based practice that has extensively demonstrated its ability to provide cost-effective solutions that may prevent and improve neurocognitive impairment (19). The “six pillars of lifestyle medicine for healthy aging” described by Jaqua et al include plant-forward diets (with an emphasis on plant-based foods), physical activity, stress management, avoiding substances such as alcohol and tobacco, restorative sleep, and maintaining social connections (19). Many of these 6 pillars correlate with risk factors identified in our study and should be considered fundamental to any prevention initiative addressing SCD.

We also found a significant association between SCDs and higher levels of childhood adversity, defined as experiencing 2 or more ACEs, independent of other risk factors. In the 2020 BRFSS, having experienced 2 or more ACEs was associated with a 69.0% increased risk of SCD later in life. This finding aligns with prior literature (11). Terry et al combined 2019 and 2020 BRFSS data and found that those with ACEs scores of 3 or more were more likely to report SCD as well as specific types of ACEs (sexual, physical, psychological, and environmental) (11). There is evidence to suggest that the stress induced by childhood adversity affects the development of executive functions, leading to decreased capacity for cognitive flexibility and working memory (20). This downstream effect of childhood adversity may partly explain the association between higher ACE scores and risk of SCD observed in our study.

We found no significant differences in SCD prevalence by race or education level in fully adjusted models. This finding contradicts prior research that demonstrated higher SCD prevalence among non-White groups and groups with lower levels of educational attainment (21). In contrast, poverty was associated with a 44.0% greater likelihood of SCD. This aligns with research conducted by Peterson on the effects of socioeconomic status across the life course and its effects on SCD (22). Peterson posits that social exposures are cumulative over time, and a high quality and quantity of diverse experiences over the life course may offer protection against SCD (22). Included in these experiences are factors such as education, income, and geographic area, which play a role in cognitive preservation (22). Additionally, lower educational attainment and income have been linked to lower health literacy — an individual’s ability to use information to make sound health-related decisions (23). Studies show that people with low health literacy skills are more likely to delay accessing care and lack a primary care physician (24). Care access plays a critical role in prevention or early identification of SCD. Policies that provide incentives to health care providers to screen for social determinants of health and that aim to increase opportunities for social and economic advancement may decrease the incidence of SCD among these socioeconomically vulnerable populations (25).

Furthermore, policies that support healthy behaviors across the life course, such as walkable built environments and interventions that address ACEs early in life, may help to prevent cognitive decline in later life (Table 4) (9). Chronic conditions such as obesity and diabetes are significant predictors of future cognitive impairment. Thus, policies targeting ACEs and chronic disease prevention could be framed as levers for HBI implementation, because they may help reduce the incidence of SCD and improve health outcomes.

**Table 4:** Examples of Policy, Environment, and Systems Changes and Interventions That Address Modifiable Risk Factors for Subjective Cognitive Decline Across the Life Course

Type of Policy or Intervention	Description
Master plans for aging	Comprehensive plans that can be adopted at the state or municipal level to address the needs of older adults and their caregivers. These multisectoral plans include strategies such as financing, infrastructure, health and social services, workforce development, housing, and transportation. Notably, these strategies could help to support healthy active living for people of all ages (26).
Adverse Childhood Experiences Response Team	The City of Manchester, New Hampshire, implemented the Adverse Childhood Experiences Response Team (ACERT) as a collaborative approach to addressing the negative effects of childhood trauma. The initiative operates through a referral mechanism that connects families to trauma-informed mental health services and social supports in the city. ACERT represents a partnership between the Manchester Police Department, YWCA-New Hampshire, and Amoskeag Health, which work collaboratively to provide assistance to families and their children who have had recent involvement with law enforcement. The program is voluntary and facilitates connections to a variety of therapies and services such as youth support groups, domestic violence services, athletic enrichment programs, home visits, and other community-based resources (31).
Built environment and infrastructure investments	Environmental and structural strategies such as increased sidewalk width, adequate lighting, increased time of pedestrian crossing lights at intersections, and intermittently spaced benches for resting can be implemented at the local level (9).
Evidence-based physical activity programs	Evidence-based programs, such as EnhanceFitness, can be funded through local or state governments to engage older adults in a more physically active lifestyle. EnhanceFitness (EF) is an effective group exercise and falls prevention program that focuses on flexibility, strength training, balance, and low-impact aerobics to improve functional abilities and independence among older adults (32). Aside from the physical benefits of exercise, this program has been proven to increase socialization, decrease depression symptoms, and reduce unplanned hospitalizations. Another evidence-based program is the Managing Overweight and Obesity for Veterans Everywhere (MOVE!) program. Approximately 78% of Veterans are overweight or obese. The VA Central Office partnered with the VA National Center for Health Promotion and Disease Prevention to develop the program. The MOVE! program was piloted between 2002 and 2004 before it was nationally implemented in 2006. The program consists of essential components including medical advisement concerning physical activity, nutrition, and behavioral health, ongoing screening and treatment of overweight or obesity, and medical documentation of weight and physical activity status (33). Veterans who participated in MOVE! were more likely to lose weight compared with those who did not participate, and the program has expanded to offer videoconferencing classes with similar outcomes (34).
Healthy diets	Diets that are associated with cognitive protection include the Mediterranean diet, the Dietary Approaches to Stop Hypertension (DASH) diet, and the Mediterranean-DASH diet Intervention for Neurodegenerative Delay (MIND). The DASH diet focuses on plant-based foods and limits the intake of short fatty acids, total fat, cholesterol, sugar, and sodium. The MIND diet incorporates elements of the Mediterranean and DASH diets. It was developed with the aim of neuroprotection and dementia prevention. The MIND diet focuses on the consumption of plant-based foods with an emphasis on berries and green vegetables while restricting red meats, sweets, dairy, and fast-fried foods. An Australian longitudinal study demonstrated a 53% decreased risk of dementia with high adherence to the MIND diet and a 35% decrease with moderate compliance (35).
Stress management	Mindfulness-based approaches, such as Acceptance and Commitment Therapy (ACT), are transdiagnostic and can help people manage stressors associated with chronic disease and pain. This type of cognitive behavioral therapy can be accessed in clinical or community settings, including via telehealth. ACT has been shown to change brain network connectivity (36).
Restorative sleep	Educational approaches focusing on sleep hygiene, combined with early screening, diagnosis, and treatment of sleep disorders, can support healthy aging. Nonpharmacological approaches such as cognitive behavioral therapy are considered first line approaches (37).
Strong social connections	Interventions using telehealth have shown promise for enabling people to maintain healthy social connections, even during the COVID-19 pandemic. Family resource centers are holistic centers for intergenerational support and kinship navigation. They can also provide intergenerational social support (38).
Avoiding misuse of substances such as alcohol and tobacco	Many examples of evidence-based interventions using theoretically grounded approaches are available in the literature. daRosa et al provide a systematic review focused on the Transtheoretical Model of behavior change for older persons (39).
Falls prevention	A Matter of Balance is a falls prevention program that has been implemented in several states. The program is designed to reduce the fear of falling and increase activity levels among older adults. Community classes can be offered both in person and virtually. The program was developed at the Roybal Center at Boston University. MaineHealth provides master trainer training sessions that prepare organizations to offer A Matter of Balance in their communities. Master trainers are responsible for teaching the Matter of Balance curriculum to coaches and providing them with guidance and support as they lead the Matter of Balance classes (40).

Beyond clinical interventions, policy and systems changes can help create environments that promote healthy behaviors among older adults, reducing their risk of SCD, falls, and associated functional difficulties (Table 4).

Older adults face unique challenges related to their social, physical, and economic environments (26). For example, retirement can impose distinctive financial limitations, and transportation becomes a barrier because adults tend to drive less frequently, or not at all, as they age (26). In response to these unique challenges, 4 states have adopted Master Plans for Aging — a collection of comprehensive state and local policy approaches to address the needs of older adults and their caregivers (Table 4) (26). For example, Massachusetts Executive Order 576 (2017) established the Governor’s Council to Address Aging, which released a blueprint outlining 28 recommendations and 67 action steps across 5 areas: caregiving, employment, housing, transportation, and innovation and technology (27). The overarching goals of this blueprint aim to ensure that aging is embedded in all policies with input from older adults, residents have the resources to live a meaningful life in the community of their choice, and people of all ages have access to health and social support (27). Not only is Massachusetts heavily tackling the lifestyle medicine pillar of social connection, but it is also attempting to lessen the economic burdens of aging, increase access to care, and promote independence and mobility in an effort to prevent chronic disease. This blueprint is an important example of a policy approach that addresses the downstream influences of healthy aging.

### Limitations

Our study has several limitations that warrant mention. First, BRFSS survey data are self-reported, which may challenge the validity of our results because of recall bias, potentially resulting in an underestimated prevalence of SCD. Second, survey participants are noninstitutionalized adults, excluding those in nursing homes or other long-term care facilities. Third, the BRFSS survey is conducted through random digit dialing of landlines and cellular telephones (12). Systematic biases may be introduced by excluding those who do not have access to a telephone. Nevertheless, the BRFSS survey includes a representative sample of the US population and is largely generalizable.

Another limitation is that the cross-sectional design of our study precludes causal inferences. The risk factors are particularly subject to a reverse causality effect in which the presence of cognitive decline could lead to feelings of depression, being more prone to frequent falls because of functional difficulties, or experiencing an increase in hours of sleep per night. Self-selection bias and recall bias also may affect the interpretation of the results.

Lastly, all models excluded observations in which data were missing for any given variable included in the analysis. Response bias may have been conferred by including only those who completed the ACEs questions. Previous studies found that participants without missing ACEs data may be more affluent compared with other participants (28). This reduced the final analytic sample size, potentially resulting in a loss of power. Methods such as multiple imputation can be used to address missing data; however, these methods have only recently been applied to ACEs because of their complexity and challenges in achieving model convergence. Houtepen et al applied a pragmatic imputation strategy in a recent longitudinal ACEs study, offering a method that could be explored in future research (29). Additionally, future research should examine mediation and moderation with respect to specific ACEs, other risk factors, and SCD.

An unexpected finding in our analysis was the lower prevalence of survey respondents who reported SCD in 2020 (8.1%) compared with prepandemic years. Historically, SCD prevalence has hovered around 11.0 %, as it did in 2019 (18). This may be due to a healthy worker effect, a form of selection bias, resulting from the COVID-19 pandemic (30). Respondents in 2020 were less likely to be employed, more likely to be working from home, and more likely to report good or better health than in 2019 (18). Participants who answered the survey in 2020 may have been relatively healthy individuals who would otherwise have been working outside the home as in prepandemic years. This argument is supported when comparing the prevalence of other chronic conditions in 2020 versus 2019. For example, the number of respondents who reported having had a prior stroke was 4.5% in 2019 compared with 3.9% in 2020 (15,18). Similar trends were observed for other diseases, such as diabetes, cancer, and chronic obstructive pulmonary disease (18). Future research should assess whether these trends continue in postpandemic years.

In conclusion, the results of our study can be used to support the HBI by informing primary and secondary prevention interventions and policies that address modifiable risk factors across the life course. Initiatives such as Master Plans for Aging, as well as those that address ACEs, can provide critical synergistic frameworks for policy, environment, and systems change that engage communities in reducing disparities faced by aging populations.
